# CRIPTO Is a Marker of Chemotherapy-Induced Stem Cell Expansion in Non-Small Cell Lung Cancer

**DOI:** 10.3389/fonc.2022.830873

**Published:** 2022-06-02

**Authors:** Federica Francescangeli, Maria Laura De Angelis, Rachele Rossi, Giovanni Sette, Adriana Eramo, Alessandra Boe, Ombretta Guardiola, Tao Tang, Shi-Cang Yu, Gabriella Minchiotti, Ann Zeuner

**Affiliations:** ^1^Department of Oncology and Molecular Medicine, Istituto Superiore di Sanità, Rome, Italy; ^2^Core Facilities, Istituto Superiore di Sanità, Rome, Italy; ^3^Stem Cell Fate Laboratory, Institute of Genetics and Biophysics “A. Buzzati Traverso”, Consiglio Nazionale delle Ricerche, Naples, Italy; ^4^Department of Stem Cell and Regenerative Medicine, Institute of Pathology and Southwest Cancer Center, Southwest Hospital, ChongQing, China; ^5^International Joint Research Center for Precision Biotherapy, Ministry of Science and Technology, ChongQing, China

**Keywords:** cancer stem cells, lung cancer, plasticity, CRIPTO, dynamic response

## Abstract

Chemotherapy is the mainstay for the treatment of non-small cell lung cancer (NSCLC). However, NSCLC cells are either intrinsically chemoresistant or rapidly develop therapy resistance. Cancer stem cells (CSCs) are widely recognized as the cell population responsible for resistance to systemic therapies, but the molecular responses of CSCs to chemotherapeutic agents are largely unknown. We identified the embryonic protein CRIPTO in stem cell-enriched spheroid cultures of adenocarcinoma (AC) and squamous cell carcinoma (SCC) derived from NSCLC surgical specimens. The CRIPTO-positive population had increased clonogenic capacity and expression of stem cell-related factors. Stemness-related properties were also obtained with forced CRIPTO expression, whereas CRIPTO downregulation resulted in cell cycle blockade and CSCs death. Cell populations positive and negative for CRIPTO expression were interconvertible, and interfering with their reciprocal equilibrium resulted in altered homeostasis of cell expansion both in spheroid cultures and in tumor xenografts. Chemotherapy treatment of NSCLC cells resulted in reduction of cell number followed by increased CRIPTO expression and selective survival of CRIPTO-positive cells. In NSCLC tumor xenografts, chemotherapeutic agents induced partial cell death and tumor stabilization followed by CRIPTO overexpression and tumor progression. Altogether, these findings indicate CRIPTO as a marker of lung CSCs possibly implicated in cancer cell plasticity and post-chemotherapy tumor progression.

## Introduction

CRIPTO, also known as teratocarcinoma-derived growth factor-1 (TDGF-1), is an extracellular glycosylphosphatidylinositol (GPI)-anchored protein (1) expressed in mouse and human embryonic stem cells (ESCs), where it regulates self-renewal and differentiation (2). CRIPTO is an obligate co-receptor for the TGF-β family members Nodal and GDF1/3 implicated in development, pluripotency and cancer (3–5). Besides its well-known role as modulator of TGF-β signaling, CRIPTO also cooperates with different pathways including WNT, AKT and NOTCH (6). CRIPTO is overexpressed in several human tumors including breast, colorectal, lung, renal, ovarian, pancreatic, prostate, gastric, bladder and esophageal cancers, melanoma and glioblastoma (7–19). CRIPTO expression has been detected by our group and others on cancer stem cells (CSCs) of colorectal, hepatocellular, esophageal carcinomas and embryonal carcinoma (20–23). In colorectal cancer (CRC) CRIPTO levels affected the size of the CSCs compartment, as CRIPTO downregulation was able to inhibit CSCs survival, tumor growth and metastasis formation (20). CRIPTO was also found to be expressed by pancreatic CSCs, where Nodal/Activin signaling proved to be essential for CSCs self-renewal, chemoresistance and *in vivo* tumorigenicity (24). In lung cancer, serum levels of soluble CRIPTO were identified as a diagnostic and prognostic marker, whereas CRIPTO expression in tumor tissues was associated with worse prognosis (16, 25–30). CRIPTO was previously proposed to mediate NSCLC resistance to the EGFR tyrosine kinase inhibitor (TKI) erlotinib (31). However, subsequent studies with the third generation EGFR TKI osimertinib showed that CRIPTO was ineffective in eliciting drug resistance, although CRIPTO serum levels mirrored patient progression and resistance to treatment (32). Altogether, these findings highlight a complex role of CRIPTO in regulating tumor stemness that deserves further investigation, also in light of potential CRIPTO targeting by molecular and immunotherapeutic strategies (33, 34). Here, we show that CRIPTO is dynamically expressed in NSCLC spheroid cultures derived from surgical specimens of lung adenocarcinoma (AC) and squamous cell carcinoma (SCC). Exogenous CRIPTO expression in NSCLC spheroids increased stemness-related gene expression, cell migration, clonogenic capacity and production of soluble CRIPTO. By contrast, CRIPTO silencing induced cell cycle arrest and subsequent CSCs extinction. Interestingly, flow cytometry separation of CRIPTO^high^ and CRIPTO^low^ cells was followed by an interconversion of these two populations, highlighting the plasticity of lung CSCs. Chemotherapy treatment of NSCLC spheroids and xenografts increased CRIPTO expression and enhanced tumor progression *in vivo* as compared to untreated tumors. According to these observations, NSCLC patients with higher CRIPTO expression had a worse prognosis. Overall, these observations indicate that CRIPTO expression identifies a dynamic population of lung CSCs that may be implicated in chemoresistance and post-chemotherapy tumor progression.

## Results

### CRIPTO Is Dynamically Expressed on Stem Cell-Enriched NSCLC Cultures

**Figure 1:**
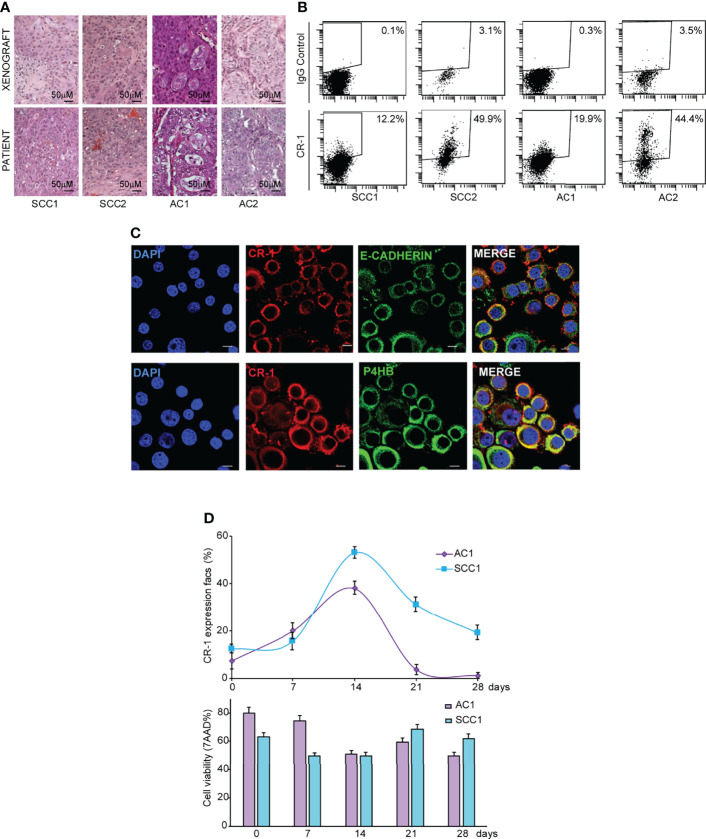
CRIPTO (CR-1) is dynamically expressed in stem cell-enriched cultures of adenocarcinoma (AC) and squamous cell carcinoma (SCC). **(A)** Hematoxylin/Eosin staining of paraffin-embedded xenograft sections obtained from two SCC and two AC spheroid cultures (upper panels) and of the parental patient tumor (lower panels), 20X magnification, scale bar 50 μm. Pictures in the upper panels are representative of xenograft sections derived from three different mice. **(B)** Flow cytometry analysis of surface CRIPTO expression on two SCC and two AC spheroid cultures derived from patients represented in **(A)** and in **Table S1**. **(C)** Immunofluorescence staining for CRIPTO in combination with E-Cadherin or Prolyl 4-Hydroxylase subunit beta (P4HB) to show respectively plasmamembrane and endoplasmic reticulum localization on AC1 cells, 60X magnification, 1.8X zoom, scale bar 10 μm. **(D)** Values obtained from flow cytometry analysis of surface CRIPTO (indicated as percentage of positive cells) in AC1 (light purple rhombus) and SCC1 (light blue square) at the indicated days. The histogram represents the percentage of FACS-positive cells for 7-amino actinomycin D (7-AAD). FACS plots are shown in **Supplemental Figure 1**.

NSCLC surgical specimens (two AC and two SCC) were cultured in serum-free medium containing EGF and basic-FGF (bFGF) to generate stem cell-enriched spheroid cultures, as previously described (35, 36). Clinical data of NSCLC patients and mutational features of NSCLC spheroids are reported in **Supplemental Table 1**. NSCLC spheroids retain the ability to produce phenocopies of the original tumors when inoculated in immunecompromised mice (**Figure 1A**) and are enriched in cells expressing stemness-related factors NANOG, SOX2 and OCT3/4 (**Supplemental Figure 1A**). We have previously demonstrated that CRIPTO is a marker of CRC CSCs and that CRIPTO expression determines CSCs-related features of CRC cells *in vitro* and *in vivo* (20). To investigate whether CRIPTO plays a role also in NSCLC CSCs, we investigated its expression in AC and SCC spheroid cultures. Flow cytometry analysis of surface CRIPTO levels showed the presence of CRIPTO^+^ cells in both AC and SCC cultures with percentages of positivity variable approximately between 12% and 50% and comparable between the two histotypes (**Figure 1B**). To analyze CRIPTO subcellular localization, we performed immunofluorescence staining of AC1 cells. Localization of CRIPTO at the plasma membrane was detected by double staining of unpermeabilized cells with CRIPTO and either E-Cadherin antibodies (**Figure 1C**, upper panels) or with Wheat Germ Agglutinin (WGA), a member of the lectin family (**Supplemental Figure 1B**). Furthermore, to analyze intracellular CRIPTO localization, and considering that CRIPTO undergoes post-translational modifications in the endoplasmic reticulum (ER) and Golgi (37, 38), cells were permeabilized and double stained with CRIPTO and the ER marker Prolyl 4-Hydroxylase subunit beta (P4HB). Confocal analysis showed that the intracellular presence of CRIPTO protein in NSCLC CSCs was mostly localized in the ER (**Figure 1C**, lower panels). Then, we analyzed CRIPTO expression over time by monitoring CRIPTO levels in AC and SCC spheroids by flow cytometry through a four-week period. Similar to what was found in CRC spheroids (20), CRIPTO positivity was subject to a dynamic regulation in both AC and SCC cells (**Figure 1D** , upper panel; FACS profiles in **Supplemental Figure 1C**). Importantly, CRIPTO levels did not depend on fluctuations of cell viability, as determined by 7-aminoactinomycin D (7-AAD) staining, as this was roughly constant throughout the culture (**Figure 1D** , lower panel). An apparent decrease of 20-50% cell viability in FACS analyses is a technical artifact due to the mechanical dissociation of spheroids before testing. Altogether, these results show for the first time that CRIPTO is expressed on NSCLC CSCs and that CRIPTO expression is dynamically regulated over time in NSCLC cultures.

### CRIPTO Expression Regulates Stemness-Related Features in NSCLC Cells

**Figure 2:**
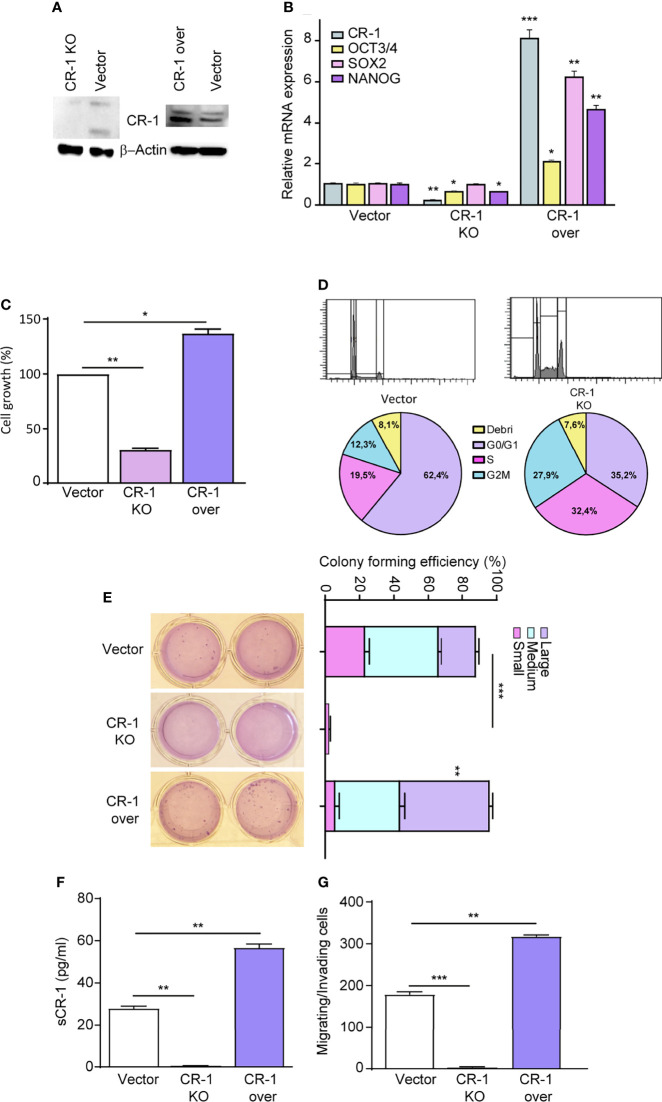
CRIPTO (CR-1) expression regulates cells proliferation and stem cell gene expression in NSCLC spheroids. **(A)** Immunoblot analysis of CRIPTO and βActin in SCC1 spheroids transduced with the empty vector (Vector), with CRIPTO shRNA vector (CRIPTO KO) or with exogenous CRIPTO (CRIPTO-over). **(B)** CRIPTO and stem cell genes (OCT3/4, SOX2, NANOG) mRNA expression in SCC1 transduced with vector, CRIPTO KO or CRIPTO-over sequences. **(C)** ATP assay performed on SCC1 transduced with the vector, CRIPTO KO or CRIPTO-over sequences, performed 4 days after transduction **(D)**. Cell cycle analysis of SCC1 transduced as above, performed 3 days after transduction. **(E)** Soft agar pictures (left; two technical replicates for each sample) and graph (right) of colony forming assay performed on control (Vector), CRIPTO KO and CRIPTO overexpressing SCC1 spheroids. The results are evaluated as colony formation in semisolid culture and expressed as normalized colony size/percentage over plated cells. **(F)** ELISA assay performed on SCC1 cells transduced with empty vector, CRIPTO KO sequences and CRIPTO overexpression vector. **(G)** Invasion/migration assay performed on vector-transduced, CRIPTO KO and CRIPTO overexpressing SCC1 cells. *P < 0.05; **P < 0.01, ***P < 0.001 by unpaired student’s t test (transduced vs vector).

To investigate whether CRIPTO plays a functional role in regulating NSCLC stemness, we modulated CRIPTO levels in SCC1 spheroids through shRNA-mediated silencing (**Figure 2A**, CRIPTO KO) or through exogenous expression of human CRIPTO driven by a lentiviral construct (**Figure 2A**, CRIPTO over). The efficacy of CRIPTO downregulation or overexpression was confirmed at the RNA level (**Figure 2B**). Then, we determined whether CRIPTO modulation affected the levels of stemness-related factors OCT3/4, SOX2 and Nanog. RT-PCR assessment of OCT3/4, SOX2 and Nanog in SCC1 spheroids showed that the expression of OCT3/4 and Nanog (but not SOX2) was downregulated in CRIPTO KO cells, while the three factors were significantly upregulated upon CRIPTO overexpression (**Figure 2B**). Then, we determined the effect of CRIPTO modulation on the growth of NSCLC spheroids. ATP measurement with the CellTiterGlo^®^ assay revealed that CRIPTO KO cultures contained a number of viable cells significantly lower than vector-transduced cultures, whereas CRIPTO overexpressing cultures contained a higher number of viable cells, indicating that CRIPTO expression affects NSCLC cell growth/viability (**Figure 2C**). To assess whether the lower number of cells in CRIPTO KO cultures was due to a proliferative arrest, we analyzed the cell cycle in SCC1 spheroids transduced with the empty vector of with CRIPTO shRNA. The CRIPTO KO sample showed an accumulation of cells in late G1/S phase and a cell cycle arrest in G2/M (**Figure 2D**), indicating that CRIPTO silencing results in cell cycle dysregulation. Then, we ought to investigate whether CRIPTO modulation was able to affect the clonogenic capacity of NSCLC cells. CRIPTO KO and CRIPTO overexpressing cells were plated in soft agar and colony formation was assessed after three weeks. While CRIPTO KO cells were virtually unable to form colonies, CRIPTO-overexpressing cells generated a slightly higher (but not significant) total number of colonies as compared to control cells. However, the amount of large colonies produced by CRIPTO overexpressing cells was significantly higher than the control, indicating a shift of the population towards a state of increased stemness (**Figure 2E**). Modulation of CRIPTO expression affected also the amount of soluble CRIPTO (sCR-1) released by NSCLC cells, as shown by enzyme-linked immunosorbent assay (ELISA) performed on the culture medium (**Figure 2F**). Finally, we analyzed whether CRIPTO modulation affected the migratory and invasive capacity of NSCLC cells. CRIPTO KO cells were unable to migrate in the transwell assay, while CRIPTO overexpressing cells showed a significantly higher migration/invasion capacity as compared to control cells (**Figure 2G**). Altogether, these observations indicate that CRIPTO plays a functional role in the determination of NSCLC stemness by enhancing stem cell-related gene expression and functional features.

### CRIPTO^high^ and CRIPTO^low^ Populations Have Different Stemness Features and Are Interconvertible Both *In Vitro* and *In Vivo*

**Figure 3:**
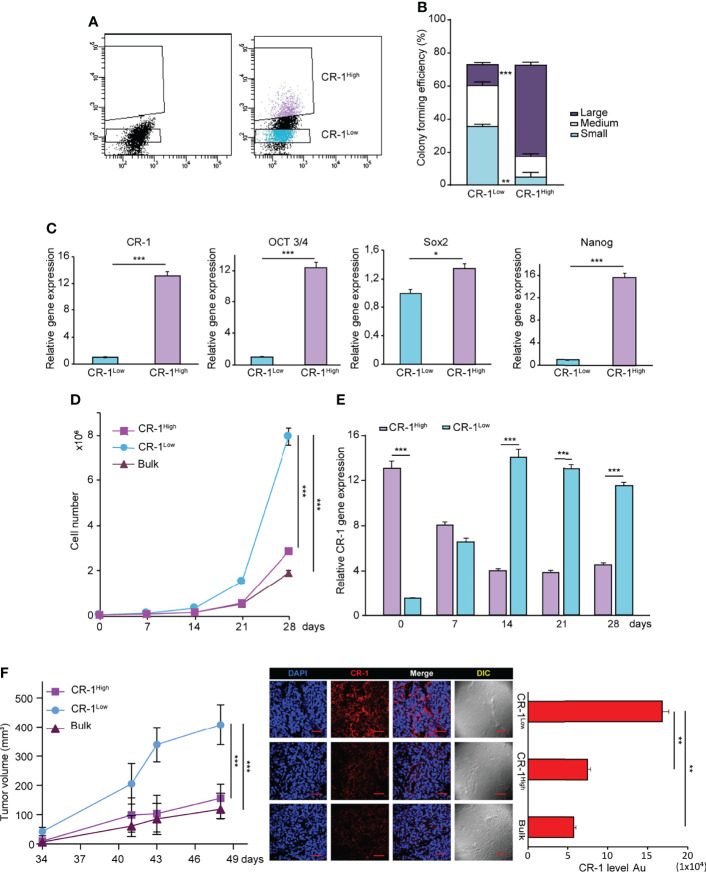
NSCLC subpopulations expressing high or low CRIPTO (CR-1) levels are interconvertible *in vitro* and *in vivo*. **(A)** FACS-based separation of CRIPTO^high^ and CRIPTO^low^ subpopulations of AC1 spheroid cultures. **(B)** Self-renewal capacity of sorted CRIPTO^high^ and CRIPTO^low^ AC1 spheroid cells, evaluated as colony formation in semisolid culture and expressed in bottom panels as normalized colony size/percentage over plated cells. **(C)** qRT-PCR analysis of CRIPTO and stem cell related genes (OCT3/4, SOX2, NANOG) performed on CRIPTO^high^ and CRIPTO^low^ subpopulations of AC1 spheroids. **(D)** Proliferation curve of CRIPTO^high^ (dark pink square) and CRIPTO^low^ (light blue dot) subpopulations or Bulk (burgundy triangle) cultures of AC1 spheroids, starting at day 0 (after sorting) and monitored at the indicated times. **(E)** qRT-PCR analysis of CRIPTO^high^ (light purple histogram) and CRIPTO^low^ (light blue histogram) subpopulations at the indicated times starting at day 0 after sorting. **(F)** Left: *In vivo* growth of tumor xenografts obtained by subcutaneous inoculation of CRIPTO^high^ (dark pink square) and CRIPTO^low^ (light blue dot) subpopulations or Bulk (burgundy triangle) cultures of AC1 spheroids, monitored at the indicated times. Results shown are the mean ± SEM of values obtained using 4 mice per group. *P < 0.05; **P < 0.01, ***P < 0.001. Middle: representative confocal images of CRIPTO (red) staining of CRIPTO^high^, CRIPTO^low^ and Bulk tumors. 40X magnification, scale bar 50 μm. Right: quantification of CRIPTO performed on 3 sets composed of 5 fields/group. **P < 0.01. AU, arbitrary units.

After assessing the effects of exogenous CRIPTO modulation, we asked whether also endogenous CRIPTO levels were related to different stemness states in NSCLC cultures. To this end, we separated CRIPTO^high^ and CRIPTO^low^ populations from AC1 spheroid cultures (**Figure 3A**) and we analyzed the clonogenic capacity and expression of stemness-related factors. After FACS sorting, CRIPTO^high^ and CRIPTO^low^ cells were seeded in soft agar and colony generation was assessed after three weeks. While the total number of colonies was similar between the two populations, CRIPTO^low^ cells generated prevalently small and medium-sized colonies. By contrast, CRIPTO^high^ cells produced predominantly large-sized colonies, indicating an increased proportion of CSCs (**Figure 3B**). RT-PCR analysis performed immediately after sorting of CRIPTO RNA levels confirmed the effective separation of CRIPTO^high^ and CRIPTO^low^ populations (**Figure 3C** , left panel). In line with higher CRIPTO expression, also the RNA levels of OCT3/4, SOX2 and Nanog were significantly increased in CRIPTO^high^ as compared to CRIPTO^low^ cells (**Figure 3C** , right panels). These results were confirmed also on SCC1 spheroid cultures (**Supplemental Figure 2**). Then, we analyzed the growth of purified CRIPTO^high^ and CRIPTO^low^ populations in liquid culture by counting dissociated spheroid cells at different time points for four weeks. Surprisingly, after the second week of culture we observed a striking proliferative increase of CRIPTO^low^ cells, while the growth of CRIPTO^high^ cells did not significantly differ from that of unseparated cultures (**Figure 3D**). Driven by this unexpected result, we analyzed CRIPTO RNA levels throughout the time of culture and we found that cells initially expressing low CRIPTO levels rapidly re-acquired CRIPTO expression and after two weeks expressed up to 13 times the initial CRIPTO RNA levels. By contrast, CRIPTO^high^ cells progressively lost excess CRIPTO expression and returned to baseline levels, which were maintained until the end of the experiment (**Figure 3E**). Finally, we investigated whether the overturning between CRIPTO^high^ and CRIPTO^low^ cells occurred also in NSCLC tumor xenografts. Freshly sorted CRIPTO^high^ and CRIPTO^low^ cells were subcutaneously inoculated in the flanks of immunocompromised mice and tumor growth was monitored through the subsequent seven weeks. In line with *in vitro* results, the growth of tumor xenografts generated by CRIPTO^high^ cells was comparable to controls, whereas tumors generated by CRIPTO^low^ cells reached a size nearly four times larger than the controls (**Figure 3F** , left panel). Accordingly, CRIPTO levels at the end of the experiment were significantly higher in the former CRIPTO^low^ population as compared to CRIPTO^high^ cells and controls (**Figure 3F** , middle and right panels) These observations indicate that CRIPTO is a metastable marker of NSCLC CSCs and highlight the plasticity of CSCs populations, particularly evident when CSCs homeostasis is perturbed.

### Chemotherapy Treatment Increases CRIPTO Expression and Growth of NSCLC Tumor Xenografts

**Figure 4:**
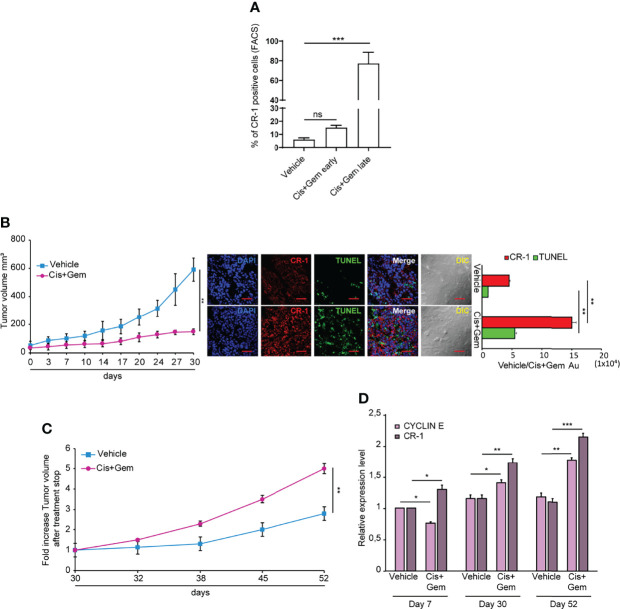
Chemotherapy treatment increases CRIPTO (CR-1) expression and tumor progression in NSCLC xenografts. **(A)** Flow cytometry analysis of CRIPTO on SCC1 cells treated with vehicle only (Vehicle) or with chemotherapeutic agents (Cis+Gem early and Cis+Gem late). Cells in the Cis+Gem samples were treated with Cisplatin 5 μM plus Gemcitabine 25 μM for 4 days then washed, replated and analyzed after 3 additional days (Cis+Gem early) or 7 additional days (Cis+Gem late). The graph shows the mean ± SD of two independent experiments. Ns, non-significant, ***P < 0.001. **(B)** Left: Xenograft volume of SCC1 spheroids cells treated with Vehicle (Vehicle, light blue square) or with Cisplatin plus Gemcitabine (Cis+Gem, Dark pink dots). Mean ± SEM, 6 mice/group. **P < 0.01 (two-tailed t test). Middle: representative confocal images of CRIPTO (red) and TUNEL (green) staining of Vehicle and Cis+Gem treated tumors. 40X magnification, scale bar 50 μm. Right: quantification of CRIPTO and TUNEL performed on 3 sets composed of 5 fields/group. **P < 0.01. AU, arbitrary units. **(C)** Tumor variation after treatment withdrawal of SCC1 spheroids treated with vehicle (Vehicle, light blue square), and Cis plus Gemcitabine (Cis+Gem, dark pink dots). Mean ± SEM, 4 mice/group. **(D)** qRT-PCR analysis of CRIPTO and Cyclin E mRNA expression of SCC1-derived xenografts, monitored during treatment and after treatment withdrawal at the indicated times. *P < 0.05; **P < 0.01, ***P < 0.001.

A proportion of NSCLC spheroids shows intrinsic resistance to chemotherapy treatment, mirroring the chemotherapy resistance typical of lung tumors (36). To investigate whether CRIPTO expression was affected by chemotherapy, we treated SCC1 spheroids with Cisplatin plus Gemcitabine (Cis+Gem) for 4 days. Then, drugs were washed out and cells were analyzed for CRIPTO expression after 3 and 7 days, respectively. Annexin V and 7-AAD stainings were added in order to exclude early-apoptotic and late-apoptotic cells, respectively (**Supplemental Figure 3**, line 2). However, cellular elements that are 7-AAD-/Annexin V- may include not only viable cells but also cellular debris, which have a low uptake of both nuclear dyes and Annexin V (39). Therefore, we have performed an analysis of the 7-AAD-/Annexin V- population for physical parameters (**Supplemental Figure 3**, line 3), allowing to discriminate viable cells from subcellular elements. In fact, cellular elements that are classified as “non-viable” in the forward scatter/side scatter (FSC/SSC) analysis include particles with very low size and variable granularity that correspond to cellular debris according to accredited flow cytometry guidelines (40). Moreover, cellular elements found in apoptotic cultures may include microvesicles released by therapy-resistant cells (41). At early times after chemotherapy treatment, CRIPTO expression increased approximately two times as compared to vehicle-treated controls, although it did not reach statistical significance. At later times, we observed a massive (~15 times) increase of CRIPTO expression in cells surviving chemotherapy treatment (**Figure 4A** and **Supplemental Figure 3**). Thus, the analysis of CRIPTO expression on cell populations purged of dead cells and debris shows that CRIPTO expression is strongly increased in surviving cancer cells that are responsible for post-chemotherapy culture recovery. To investigate whether the same phenomenon occurred *in vivo*, we generated subcutaneous xenografts with SCC1 spheroids and we treated mice with Cis+Gem, monitoring tumor volume for 30 days. NSCLC tumor xenografts responded to chemotherapy treatment with a growth slowdown and stabilization for the duration of the experiment (**Figure 4B** , left panel). Notably, immunofluorescence analysis of tumor sections at the end of the experiment showed an increased amount of dead cells in treated tumors (as assessed by TUNEL assay) and a strong increase of CRIPTO expression (**Figure 4B** , middle and right panels), indicating that chemotherapy induces CRIPTO expression also *in vivo* in NSCLC xenografts. Importantly, monitoring tumor growth after treatment cessation showed that chemotherapy-treated xenografts underwent rapid progression, reaching five times the starting size as compared to the two times increase of untreated tumors (**Figure 4C**). We analyzed the levels of cyclin E (as a marker of cell proliferation) and of CRIPTO in tumor xenografts harvested at different times of treatment, i.e. after the first week of treatment, at treatment stop and after three additional weeks of tumor growth. Interestingly, we found that during the first phase of chemotherapy cyclin E levels were lower than controls, according to tumors’ growth slowdown. Subsequently, cyclin E expression started to increase around the end of treatment, possibly due to the thriving of chemoresistant cells, and further increased during tumor progression. CRIPTO levels showed a slightly different trend, as they started to raise shortly after treatment start and progressively increased at treatment stop and during tumor progression (**Figure 4D**). These results show that CRIPTO levels strongly increased following treatment with anticancer drugs both *in vitro* and *in vivo*. The increase of CRIPTO expression mirrored chemotherapy-induced tumor progression in NSCLC xenografts and may reflect a selective survival of chemoresistant CSCs. In line with these results, an analysis of NSCLC gene expression datasets showed that higher CRIPTO expression correlated with worse prognosis in both AC and SCC (**Supplemental Figure 4**).

## Discussion

CRIPTO is a multifunctional signaling molecule with a well-defined role in embryonic development and pluripotent stem cells (4), and an emerging function in tissue regeneration (42–44). Despite a large set of literature describing CRIPTO overexpression in a variety of tumors, the role of CRIPTO in cancer is less defined. Indeed, several studies support a correlation between CRIPTO levels and worse prognosis in multiple cancers, but the mechanistic role of CRIPTO in cancer cell regulation is largely unknown. We have previously characterized CRIPTO as a functional marker of CRC stem cells, involved in the regulation of self-renewal, tumorigenesis and metastatic ability (20). In this manuscript we demonstrate, for the first time to our knowledge, that CRIPTO is expressed on NSCLC CSCs and is responsible for the regulation of stemness-associated features. Furthermore, our results showed that CRIPTO is involved in NSCLC CSCs plasticity, as separation of CRIPTO^high^ and CRIPTO^low^ cells resulted in a rapid modification of CRIPTO levels in the two subpopulations and in a functional response in terms of tumor cell expansion. In fact, while CRIPTO^high^ cells shortly returned to baseline CRIPTO levels and proliferation state, CRIPTO^low^ cells reacted to the perturbation with a strong increase of CRIPTO RNA and a corresponding proliferative burst observed both in tumor spheroids and xenografts. Interestingly, previous studies on ESCs showed that separation of CRIPTO^high^ and CRIPTO^low^ cells was followed by a return to equilibrium of both populations, and that fluctuation of Cripto protein levels do not reflect changing in the RNA levels (2). Therefore, the strong expansion of NSCLC cells/xenografts in response to the depletion of CRIPTO^high^ cells (that likely correspond to cells with increased stemness features) may be viewed as a form of altered homeostasis typical of tumors. In fact, while normal tissues usually respond to perturbations with a return to homeostasis, tumors often respond to cytotoxic challenges by increasing their survival cues (i.e. by undergoing epithelial-mesenchymal transition) and preparing for subsequent regrowth, according to the therapy-induced cancer repopulation model (41, 45, 46). In particular, in several tumors chemotherapy treatment has been shown to result in an increase in stem cell content (46–48). Specifically, NSCLC stem cells have been demonstrated to survive chemotherapy (35, 36, 49). Furthermore, shorter progression-free survival was observed in chemotherapy-treated NSCLC patients expressing the CSCs marker CD133 (49). In support of these observations, clinical studies on NSCLC patients showed an accelerated tumor regrowth after induction chemotherapy (50, 51), which may be likely due to a reactive expansion of the CSCs compartment. Further studies will be needed to demonstrate a causal role of CRIPTO in NSCLC and in post-chemotherapy tumor cell repopulation. The findings reported herein and our previous data (20) suggest that CRIPTO may act as a molecular rheostat that reacts to depletion mechanisms and preserves the CSC population. Definitive implication of CRIPTO in NSCLC relapse and progression may lead to targeted therapeutic approaches aimed at interfering with post-chemotherapy CSCs expansion and to the use of CRIPTO as a marker/readout of chemotherapy-induced CSC expansion.

## Materials and Methods

### NSCLC Stem Cells Isolation and Spheroid Cultures

NSCLC cells were isolated as previously described from surgically resected tumor samples through selective culture in serum-free medium containing EGF 20 ng/ml and bFGF 10 ng/ml (PeproTech, London, UK) (35). Nontreated polystyrene flasks (Thermo Fischer Scientific, Waltham, MA, USA) were used to reduce cell adherence and to support the growth of lung CSCs as multicellular spheroids. Regular thawing of early-passage cells was carried out to avoid the accumulation of culture-related changes.

### Flow Cytometry and Cell Cycle Analysis

Dissociated spheroid cells were labelled with primary non-conjugated rabbit polyclonal antibody against CRIPTO, produced as described in (52) for 1 h on ice and with donkey anti-rabbit IgG Alexa Fluor^®^647-conjugated secondary antibody (#A31573,Thermo Fisher) for 30 min on ice, then sorted with a FACSAria (BD Biosciences, San Jose, CA, USA) or analyzed with a FACSCanto flow cytometer equipped with a DIVA software (BD Biosciences). 10 μg/ml 7-aminoactinomycin D (Sigma-Aldrich) was always added for dead cell exclusion. FITC-conjugated Annexin V was purchased from E-Bioscience/Thermo Fisher Scientific. The cell cycle status of NSCLC spheroids was assessed by staining dissociated spheroids with 50 μg/ml propidium iodide dissolved in buffer 0.1% trisodium citrate, 9.65 mM NaCl, 0.1% NP40, 200 μg/ml RNAse for 1 h at room temperature. Analysis of debris/apoptotic cells was performed by gating the desired area of the plot on the basis of physical parameters and subsequent fluorescence analysis on a linear scale.

### Immunofluorescence Analyses

For immunofluorescence (IF) analyses in **Figure 1C** and **Supplemental Figure 1B**, AC1 cells were fixed in 2% paraformaldehyde (PFA) and either permeabilized in 0.5% Triton X-100 (Bio-Rad Laboratories, Hercules, CA, USA) for analyzing intracellular localization of CRIPTO, or not permeabilized. Fixed cells were then incubated overnight (ON) at 4°C with rabbit polyclonal CRIPTO antibodies, and either mouse monoclonal anti-P4HB antibody (#ab2792, Abcam) or anti-mouse E-cadherin (Clone ECCD-2) (#M108, Takara) followed by incubation with the appropriate secondary antibodies for 1 hour at room temperature in the dark. For double staining with Wheat Germ Agglutinin (WGA), unpermeabilized cells were first incubated with anti CRIPTO antibodies (ON at 4°C) and Alexa Fluor^®^647-conjugated donkey anti-rabbit IgG, and then with biotinylated WGA (#B-1025-5, Vector Laboratories) followed by incubation with Streptavidin, Alexa Fluor 488 Conjugate (#S32354, Invitrogen) for 30 min at room temperature. Cells were counterstained with DAPI. For IF analyses in **Supplemental Figure 1B**, cells were fixed in 2% PFA and permeabilized in 0.1% Triton X-100 then incubated overnight at 4°C with polyclonal goat anti-NANOG, polyclonal goat anti-SOX2 and polyclonal goat anti-OCT3/4, (#AF1997, #AF2018, #AF1759 R&D System). After two washes in PBS, cells were incubated with appropriate secondary antibodies (donkey anti-rabbit IgG Alexa Fluor^®^647-conjugated, donkey anti-goat IgG Alexa Fluor^®^647-conjugated (#A32849) and donkey anti-goat IgG Alexa Fluor^®^555-conjugated (#A21432)) for 30 min at room temperature in the dark, stained for 15 min with 4′,6-diamidino-2-fenilindole (DAPI, #D1306) and subsequently mounted with ProLong Gold Antifade (Agilent Technologies). For IF analysis of xenograft sections, tumour tissue samples were collected in Optimal Cutting Temperature (OCT compound), frozen on dry ice and stored at − 80°C until further use. Five micrometers tissue sections were cut with a cryostat and mounted on coverslips. Tissue sections were fixed with 2% paraformaldehyde, permeabilized with 0.1% Triton X-100/PBS, quenched with 1 M glycine in PBS and incubated overnight at 4°C with primary non-conjugated rabbit polyclonal antibody against CRIPTO. After washing in PBS, sections were incubated with a donkey anti-rabbit IgG Alexa Fluor^®^647-conjugated secondary antibody for 45 minutes at room temperature in the dark. Nuclei were counterstained with DAPI for 15 minutes at room temperature. All antibodies were dissolved in PBS containing 3% bovine serum albumin (BSA), 3% fetal bovine serum (FBS), 0.001% NaN3 and 0.1% Triton X-100. For *in situ* apoptosis detection in tumor xenograft sections, TUNEL reaction was performed using the *In Situ* Cell Death Detection kit (Roche Molecular Biochemicals) according to the manufacturer’s instructions. Slides were mounted with Prolong-Gold Antifade (Thermo Fisher) and analyzed using a Zeiss LSM900 Confocal microscope equipped with a 40X oil immersion objective.

### Lentiviral Infection

For stable CRIPTO silencing, the lentiviral pLKO.1 silencing vector containing shRNA 4890, that targets the coding sequence of CRIPTO was purchased from Sigma-Aldrich (20). pLKO.1 containing a non-targeting sequence was used as a control. For exogenous CRIPTO expression, human CRIPTO cDNA was subcloned into the Pallino βA vector as previously described (53).

### Western Blotting

Cell lysates were obtained from approximately 2.5 × 10^5^ spheroid cells by incubation of cell pellets in 1% NP40 lysis buffer (20 mM Tris HCl pH 7.2, 200 mM NaCl, 1% NP40) supplemented with protease inhibitor cocktail and phosphatase inhibitor cocktails I and II (all from Sigma-Aldrich). Lysate concentrations were determined by the Bradford assay (Bio-Rad Laboratories) and equal amounts of proteins were loaded on a 4-12% precast gel (Invitrogen) and transferred to nitrocellulose membranes. Blots were blocked with TBST 5% nonfat dry milk and incubated overnight at 4°C with primary rabbit polyclonal antibody against CRIPTO, then incubated for 45 min with secondary HRP-conjugated antibodies (GE Healthcare, Uppsala, Sweden) dissolved in TBST 1% BSA. Monoclonal anti-β-actin was from Sigma-Aldrich. Chemiluminescent signals were detected with Super Signal West Pico (Pierce Pierce, Waltham, MA, USA).

### Quantitative Real-Time PCR

Total RNA was extracted with TRIzol (Invitrogen) following manufacturer’s instructions. 1 μg of RNA were reverse transcribed with M-MLV reverse transcriptase (Thermo Fisher) and cDNA were used as template in the PCR reactions. Specific primers were used for CRIPTO (Forward: 5’-ACAGAACCTGCTGCCTGAAT-3’, Reverse: 5’-ATCACAGCCGGGTAGAAATG-3’) while a TaqMan assay (Integrated DNA Technologies, IDT, Coralville, USA) was used for NANOG (Hs.PT.58.21480849), OCT3/4 (Hs.PT.58.14494169.g) and SOX2 (Hs.PT.58.237897.g). Normalization was performed using GAPDH (Forward: 5’-TGGAAGATGGTGATGGGATT-3’, Reverse: 5’-GAGTCAACGGATTTGGTCGT-3’) as reference for CRIPTO and using β-actin (Hs.PT.39a.22214847) as reference for NANOG, OCT3/4 and SOX2. Values are expressed in terms of 2-ΔΔCT where ΔΔCT=ΔCTsample−ΔCTcalibrator. ΔCT is the difference in threshold cycles between the specific RNA and reference amplicons, and CT is a parameter given by StepOne Plus Real-Time PCR software by negative correlation with an internal reference dye (ROX).

### Proliferation Assays

For proliferation assays shown in **Figure 2C**, spheroids were dissociated into single cells and plated in 96 well plates (3,5 x10^3^ cells per well). After 24 hrs, cells were treated with chemotherapeutic agents for 4 days in a humidified atmosphere at 37°C, 5% CO_2_. Cell viability was determined with the CellTiterGlo^®^ viability assay (Promega, Madison, WI, USA) with a DTX880 multimode microplate reader (Beckman Coulter). For the experiment shown in **Figure 3D**, cells were plated immediately after sorting in 100 μl at a concentration of 10^5^/ml and manually counted once a week upon spheroid dissociation with TrypLE^®^ Express. At every time point, after counting cells were re-plated at the initial concentration and after four weeks cell proliferation was calculated as the ratio between the number of counted cells and the number of plated cells, multiplied for the dilution factor.

### Clonogenicity Assay

For clonogenic assays, dissociated spheroids were plated in triplicate at 500 cells/well suspended in 0.3% agarose over a layer of 0.4% agarose. Plates were incubated in a 5% CO_2_ humidified incubator at 37°C and colony counts were performed 21 days after plating. Colonies were stained with crystal violet (0.1% in 10% MetOH) and classified as follows: small 30–60 μm, medium 60–90 μm and large >90 μm. Data represent the percentage of colony numbers relative to plated cells.

### Immunohistochemistry

Tissues were fixed in 4% PFA followed by dehydration, paraffin embedding, sectioning, and standard H&E staining. Images were acquired on a Zeiss Axio Scope.A1 Microscope equipped with 20X objective.

### Migration/Invasion Assay

1 × 10^4^ control or CRIPTO-interfered NSCLC spheroids were suspended in 200 μl of non-supplemented stem cell medium and plated into the upper wells of Matrigel-coated modified Boyden Chambers containing porous 8 μm diameter polycarbonate membranes (Costar Scientific Corporation, Cambridge, MA, USA). Lower wells contained 500 μl of stem cell medium supplemented with 20 ng/ml EGF and 10 ng/ml bFGF. After 3 days, the cells in the upper wells were removed, whereas the cells that migrated to the lower wells were fixed, stained with DAPI in PBS 1% NP40 for 5 min and counted under a fluorescence Zeiss Axio Scope.A1 microscope equipped with a 10X objective. The number of migrated cells was quantified with the software ZEN 2.6 (blue edition).

### ELISA Assay

ELISA–based assay was performed as previously described (42). Sheep anti-mouse CRIPTO Ab (R&D System, #AF1538) and mouse CRIPTO biotinylated Ab (R&D System, #BAF 1538) were used for coating and detection, respectively. Plates were incubated with avidin/streptavidin complex conjugated with horse-radish peroxidase (Vectastain elite ABC kit, Vector Laboratories) and the signals visualized with o-phenylenediamine peroxidase substrate (OPD, Sigma-Aldrich). The relative absorbance was read at 490 nm on a Benchmark microplate reader (Bio-Rad Laboratories).

### *In Vivo* Experiments

All animal procedures were performed according to the Italian National animal experimentation guidelines (D.L.116/92) upon approval of the experimental protocol by the Italian Ministry of Health’s Animal Experimentation Committee (DM n. 292/2015 PR 23/4/2015). For tumorigenesis assays, female 6–8 weeks old NOD.Cg-Prkdcscid Il2rgtm1Wjl/SzJ (NSG) mice (The Jackson Laboratory) were subcutaneously injected with 5x10^5^ dissociated spheroid cells in 100 μl 1:1 PBS/Matrigel (BD Biosciences). Tumor volume was evaluated by using an external digital caliper and volumes were calculated using the following formula: π/6 x d2 x D, where d and D represent shorter and longer tumor measurements, respectively. For CRIPTO expression experiments, 10^5^ sorted CRIPTO^low^, CRIPTO^high^ and bulk NSCLC spheroids were injected subcutaneously in the flank of NSG mice and measured two times a week as described above. For drug treatment experiments, 5 × 10^5^ NSCLC spheroid cells were injected subcutaneously in the flank of NSG mice, in 100 μl 1:1 PBS/Matrigel (BD Biosciences). Tumors were measured as described above and drug treatments started when tumor volume reached 50–100 mm^3^. Mice were randomized in control and treatment group and treated with chemotherapeutic agent combinations of cisplatin 3 mg/kg and gemcitabine 60 mg/kg intraperitoneally biweekly. Control animals were treated with vehicle only. Tumor growth was measured two times a week. For experiments shown in **Figures 3D** and **4B**, for each variable (CRIPTO expression and chemotherapy effect, respectively) we set as minimal relevant percent difference between groups K=50% corresponding to a Cohen delta=K1 = 2. This choice, as analyzed by G Power software at p=0.05 and statistical power=0.80, corresponds to a minimal sample size N=6 mice per group. Animals were euthanized according to the national Animal Welfare Guidelines. Gemcitabine and cisplatin were both from Selleckchem (Houston, TX, USA).

### Statistical Analysis

Statistical analyses of *in vitro* and *in vivo* experiments were performed using GraphPad Prism version 4.0 for Windows (GraphPad Software) with non-paired student’s-t test. Results are presented as the mean ± SD or mean ± SEM where appropriate. Statistical significance is expressed as *P < 0.05, **P < 0.01 and ***P < 0.001. Statistical analyses of NSCLC databases were performed by IBM SPSS 20.0 and GraphPad Prism 6.0. Results were expressed as mean ± SEM and *p* < 0.05 was considered to be statistically significant. The GSE41271 dataset (containing mainly lung AC) and the TCGA dataset (www.cbioportal.org) (for lung SCC) were included in the analysis.

## Data Availability Statement

The original contributions presented in the study are included in the article/**Supplementary Material**. Further inquiries can be directed to the corresponding authors.

## Ethics Statement

The studies involving human participants were reviewed and approved by the ethics committee on human experimentation of the Istituto Superiore di Sanità (authorization no. CE5ISS 09/282) and of Istituto di Ricovero e Cura a Carattere Scientifico (IRCCS) Lazio (authorization no. 1558/21). The patients/participants provided their written informed consent to participate in this study. The animal study was reviewed and approved by the Italian Ministry of Health’s Animal Experimentation Committee (DM n. 292/2015 PR 23/4/2015).

## Author Contributions

FF, MLDA, RR, AB, AE, OG, and GS performed experiments and analyzed data. S-CY and TT performed database analyses. GM provided essential expertise. AZ and FF conceived the study and wrote the manuscript. All authors read and approved the final manuscript.

## Funding

This work was supported by the Italian Association for Cancer Research (AIRC) with AIRC Investigator Grant #20744 to AZ and AIRC Investigator Grant #20736 to GM.

## Conflict of Interest

The authors declare that the research was conducted in the absence of any commercial or financial relationships that could be construed as a potential conflict of interest.

## Publisher’s Note

All claims expressed in this article are solely those of the authors and do not necessarily represent those of their affiliated organizations, or those of the publisher, the editors and the reviewers. Any product that may be evaluated in this article, or claim that may be made by its manufacturer, is not guaranteed or endorsed by the publisher.

## References

[1] Minchiotti G Parisi S Liguori G Signore M Lania G Adamson ED. Membrane-Anchorage of Cripto Protein by Glycosylphosphatidylinositol and its Distribution During Early Mouse Development. Mech Dev (2000) 90(2):133–42. doi: 10.1016/s0925-4773(99)00235-x 10640699

[2] Fiorenzano A Pascale E D'Aniello C Acampora D Bassalert C Russo F. Cripto is Essential to Capture Mouse Epiblast Stem Cell and Human Embryonic Stem Cell Pluripotency. Nat Commun (2016) 7:12589. doi: 10.1038/ncomms12589 27586544 PMC5025790

[3] Topczewska JM Postovit LM Margaryan NV Sam A Hess AR Wheaton WW. Embryonic and Tumorigenic Pathways Converge *via* Nodal Signaling: Role in Melanoma Aggressiveness. Nat Med (2006) 12(8):925–32. doi: 10.1038/nm1448 16892036

[4] Strizzi L Bianco C Normanno N Salomon D. Cripto-1: A Multifunctional Modulator During Embryogenesis and Oncogenesis. Oncogene (2005) 24(37):5731–41. doi: 10.1038/sj.onc.1208918 16123806

[5] Postovit LM Margaryan NV Seftor EA Kirschmann DA Lipavsky A Wheaton WW. Human Embryonic Stem Cell Microenvironment Suppresses the Tumorigenic Phenotype of Aggressive Cancer Cells. Proc Natl Acad Sci U.S.A. (2008) 105(11):4329–34. doi: 10.1073/pnas.0800467105 PMC2393795 18334633

[6] Freeman DW Rodrigues Sousa E Karkampouna S Zoni E Gray PC Salomon DS. Whence CRIPTO: The Reemergence of an Oncofetal Factor in 'Wounds' That Fail to Heal. Int J Mol Sci (2021) 22(18):10164. doi: 10.3390/ijms221810164 34576327 PMC8472190

[7] De Luca A Lamura L Strizzi L Roma C D'Antonio A Margaryan N. Expression and Functional Role of CRIPTO-1 in Cutaneous Melanoma. Br J Cancer (2011) 105(7):1030–8. doi: 10.1038/bjc.2011.324 PMC3185940 21863025

[8] Friess H Yamanaka Y Buchler M Kobrin MS Tahara E Korc M. Cripto, a Member of the Epidermal Growth Factor Family, is Over-Expressed in Human Pancreatic Cancer and Chronic Pancreatitis. Int J Cancer (1994) 56(5):668–74. doi: 10.1002/ijc.2910560511 8314343

[9] Liu Y Wang J Yang T Liu R Xu Y. Overexpression Levels of Cripto-1 Predict Poor Prognosis in Patients With Prostate Cancer Following Radical Prostatectomy. Oncol Lett (2019) 18(3):2584–91. doi: 10.3892/ol.2019.10555 PMC6676627 31452743

[10] Mahmoudian RA Abbaszadegan MR Forghanifard MM Moghbeli M Moghbeli F Chamani J. Biological and Clinicopathological Significance of Cripto-1 Expression in the Progression of Human ESCC. Rep Biochem Mol Biol (2017) 5(2):83–90. doi: 10.1016/j.jocit.2017.04.036 28367468 PMC5346274

[11] Panico L D'Antonio A Salvatore G Mezza E Tortora G De Laurentiis M. Differential Immunohistochemical Detection of Transforming Growth Factor Alpha, Amphiregulin and CRIPTO in Human Normal and Malignant Breast Tissues. Int J Cancer (1996) 65(1):51–6. doi: 10.1002/(SICI)1097-0215(19960103)65:1<51::AID-IJC9>3.0.CO;2-0 8543395

[12] Pilgaard L Mortensen JH Henriksen M Olesen P Sorensen P Laursen R. Cripto-1 Expression in Glioblastoma Multiforme. Brain Pathol (2014) 24(4):360–70. doi: 10.1111/bpa.12131 PMC8029290 24521322

[13] Sato J Karasawa H Suzuki T Nakayama S Katagiri M Maeda S. The Function and Prognostic Significance of Cripto-1 in Colorectal Cancer. Cancer Invest (2020) 38(4):214–27. doi: 10.1080/07357907.2020.1741604 32157913

[14] Wang JH Wei W Xu J Guo ZX Xiao CZ Zhang YF. Elevated Expression of Cripto-1 Correlates With Poor Prognosis in Hepatocellular Carcinoma. Oncotarget (2015) 6(33):35116–28. doi: 10.18632/oncotarget.5057 PMC4741514 26375669

[15] Wei B Jin W Ruan J Xu Z Zhou Y Liang J. Cripto-1 Expression and its Prognostic Value in Human Bladder Cancer Patients. Tumour Biol (2015) 36(2):1105–13. doi: 10.1007/s13277-014-2695-1 25326807

[16] Xu C Yuan Q Hu H Wang W Zhang Q Li L. Expression of Cripto-1 Predicts Poor Prognosis in Stage I non-Small Cell Lung Cancer. J Cell Mol Med (2020) 24(17):9705–11. doi: 10.1111/jcmm.15518 PMC7520286 32697011

[17] Xue YJ Chen SN Chen WG Wu GQ Liao YF Xu JB. Cripto-1 Expression in Patients With Clear Cell Renal Cell Carcinoma is Associated With Poor Disease Outcome. J Exp Clin Cancer Res (2019) 38(1):378. doi: 10.1186/s13046-019-1386-6 31455359 PMC6712621

[18] Yoon HJ Hong JS Shin WJ Lee YJ Hong KO Lee JI. The Role of Cripto-1 in the Tumorigenesis and Progression of Oral Squamous Cell Carcinoma. Oral Oncol (2011) 47(11):1023–31. doi: 10.1016/j.oraloncology.2011.07.019 21824804

[19] Zhang JG Zhao J Xin Y. Significance and Relationship Between Cripto-1 and P-STAT3 Expression in Gastric Cancer and Precancerous Lesions. World J Gastroenterol (2010) 16(5):571–7. doi: 10.3748/wjg.v16.i5.571 PMC2816268 20128024

[20] Francescangeli F Contavalli P De Angelis ML Baiocchi M Gambara G Pagliuca A. Dynamic Regulation of the Cancer Stem Cell Compartment by Cripto-1 in Colorectal Cancer. Cell Death Differ (2015) 22(10):1700–13. doi: 10.1038/cdd.2015.19 PMC4563784 26343543

[21] Liu Q Cui X Yu X Bian BS Qian F Hu XG. Cripto-1 Acts as a Functional Marker of Cancer Stem-Like Cells and Predicts Prognosis of the Patients in Esophageal Squamous Cell Carcinoma. Mol Cancer (2017) 16(1):81. doi: 10.1186/s12943-017-0650-7 28431580 PMC5399850

[22] Lo RC Leung CO Chan KK Ho DW Wong CM Lee TK. Cripto-1 Contributes to Stemness in Hepatocellular Carcinoma by Stabilizing Dishevelled-3 and Activating Wnt/beta-Catenin Pathway. Cell Death Differ (2018) 25(8):1426–41. doi: 10.1038/s41418-018-0059-x PMC6113239 29445127

[23] Watanabe K Meyer MJ Strizzi L Lee JM Gonzales M Bianco C. Cripto-1 is a Cell Surface Marker for a Tumorigenic, Undifferentiated Subpopulation in Human Embryonal Carcinoma Cells. Stem Cells (2010) 28(8):1303–14. doi: 10.1002/stem.463 PMC3069615 20549704

[24] Lonardo E Hermann PC Mueller MT Huber S Balic A Miranda-Lorenzo I. Nodal/Activin Signaling Drives Self-Renewal and Tumorigenicity of Pancreatic Cancer Stem Cells and Provides a Target for Combined Drug Therapy. Cell Stem Cell (2011) 9(5):433–46. doi: 10.1016/j.stem.2011.10.001 22056140

[25] Park KS Moon YW Raffeld M Lee DH Wang Y Giaccone G. High Cripto-1 and Low miR-205 Expression Levels as Prognostic Markers in Early Stage non-Small Cell Lung Cancer. Lung Cancer (2018) 116:38–45. doi: 10.1016/j.lungcan.2017.12.010 29413049 PMC8057110

[26] Wei Y Jiang J Wang C Zou H Shen X Jia W. Prognostic Value of Cripto-1 Expression in non-Small-Cell Lung Cancer Patients: A Systematic Review and Meta-Analysis. biomark Med (2020) 14(4):317–29. doi: 10.2217/bmm-2019-0159 32134335

[27] Xu CH Cao L Wei Y Yu LK. Serum Cripto-1 as a Clinical Marker for Lung Cancer. Int J Biol Markers (2015) 30(4):e369–73. doi: 10.5301/jbm.5000155 26109366

[28] Xu CH Chi CZ Zhang Q Wang YC Wang W Yuan Q. Diagnostic and Prognostic Value of Serum Cripto-1 in Patients With non-Small Cell Lung Cancer. Clin Respir J (2018) 12(10):2469–74. doi: 10.1111/crj.12793 29570945

[29] Xu CH Sheng ZH Hu HD Hao KK Wang QB Yu LK. Elevated Expression of Cripto-1 Correlates With Poor Prognosis in non-Small Cell Lung Cancer. Tumour Biol (2014) 35(9):8673–8. doi: 10.1007/s13277-014-2039-1 24870591

[30] Zhang H Zhang B Gao L Zhang L Zhu K Cheng R. Clinical Significance of Cripto-1 Expression in Lung Adenocarcinoma. Oncotarget (2017) 8(45):79087–98. doi: 10.18632/oncotarget.15761 PMC5668022 29108289

[31] Park K-S Raffeld M Moon YW Xi L Bianco C Pham T. CRIPTO1 Expression in EGFR-Mutant NSCLC Elicits Intrinsic EGFR-Inhibitor Resistance. J Clin Invest (2014) 124(7):3003–15. doi: 10.1172/JCI73048 PMC4071378 24911146

[32] Chen V Iwama E Kim I-K Giaccone G. Serum CRIPTO Does Not Confer Drug Resistance Against Osimertinib But is an Indicator of Tumor Burden in non-Small Cell Lung Cancer. Lung Cancer (2020) 145:48–57. doi: 10.1016/j.lungcan.2020.04.032 32408132

[33] Arnouk H Yum G Shah D. Cripto-1 as a Key Factor in Tumor Progression, Epithelial to Mesenchymal Transition and Cancer Stem Cells. Int J Mol Sci (2021) 22(17):9280. doi: 10.3390/ijms22179280 34502188 PMC8430685

[34] Ishii H Afify SM Hassan G Salomon DS Seno M. Cripto-1 as a Potential Target of Cancer Stem Cells for Immunotherapy. Cancers (2021) 13(10):2491. doi: 10.3390/cancers13102491 34065315 PMC8160785

[35] Eramo A Lotti F Sette G Pilozzi E Biffoni M Di Virgilio A. Identification and Expansion of the Tumorigenic Lung Cancer Stem Cell Population. Cell Death Differ (2008) 15(3):504–14. doi: 10.1038/sj.cdd.4402283 18049477

[36] Zeuner A Francescangeli F Contavalli P Zapparelli G Apuzzo T Eramo A. Elimination of Quiescent/Slow-Proliferating Cancer Stem Cells by Bcl-XL Inhibition in non-Small Cell Lung Cancer. Cell Death Differ (2014) 21(12):1877–88. doi: 10.1038/cdd.2014.105 PMC4227145 25034785

[37] Blanchet MH Le Good JA Mesnard D Oorschot V Baflast S Minchiotti G. Cripto Recruits Furin and PACE4 and Controls Nodal Trafficking During Proteolytic Maturation. EMBO J (2008) 27(19):2580–91. doi: 10.1038/emboj.2008.174 PMC2567404 18772886

[38] Constam D. Riding Shotgun: A Dual Role for the Epidermal Growth Factor-Cripto/FRL-1/Cryptic Protein Cripto in Nodal Trafficking. Traffic (2009) 10(7):783–91. doi: 10.1111/j.1600-0854.2009.00874.x 19302412

[39] Jiang L Tixeira R Caruso S Atkin-Smith GK Baxter AA Paone S. Monitoring the Progression of Cell Death and the Disassembly of Dying Cells by Flow Cytometry. Nat Protoc (2016) 11(4):655–63. doi: 10.1038/nprot.2016.028 26938116

[40] Cossarizza A Chang HD Radbruch A Akdis M Andrä I Annunziato F. Guidelines for the Use of Flow Cytometry and Cell Sorting in Immunological Studies. Eur J Immunol (2017) 47(10):1584–797. doi: 10.1002/eji.201970107 PMC9165548 29023707

[41] Corsi F Capradossi F Pelliccia A Briganti S Bruni E Traversa E. Apoptosis as Driver of Therapy-Induced Cancer Repopulation and Acquired Cell-Resistance (CRAC): A Simple *In Vitro* Model of Phoenix Rising in Prostate Cancer. Int J Mol Sci (2022) 23(3):1152. doi: 10.3390/ijms23031152 35163077 PMC8834753

[42] Guardiola O Lafuste P Brunelli S Iaconis S Touvier T Mourikis P. Cripto Regulates Skeletal Muscle Regeneration and Modulates Satellite Cell Determination by Antagonizing Myostatin. Proc Natl Acad Sci U.S.A. (2012) 109(47):E3231–40. doi: 10.1073/pnas.1204017109 PMC3511144 23129614

[43] Iavarone F Guardiola O Scagliola A Andolfi G Esposito F Serrano A. Cripto Shapes Macrophage Plasticity and Restricts EndMT in Injured and Diseased Skeletal Muscle. EMBO Rep (2020) 21(4):e49075. doi: 10.15252/embr.201949075 32107853 PMC7132341

[44] Karkampouna S van der Helm D van Hoek B Verspaget HW Goumans MJ Coenraad MJ. Oncofetal Protein CRIPTO Regulates Wound Healing and Fibrogenesis in Regenerating Liver and is Associated With the Initial Stages of Cardiac Fibrosis. Cells (2021) 10(12):3325. doi: 10.3390/cells10123325 34943832 PMC8699799

[45] Shibue T Weinberg RA. EMT. CSCs, and Drug Resistance: The Mechanistic Link and Clinical Implications. Nat Rev Clin Oncol (2017) 14(10):611–29. doi: 10.1038/nrclinonc.2017.44 PMC5720366 28397828

[46] D'Alterio C Scala S Sozzi G Roz L Bertolini G. Paradoxical Effects of Chemotherapy on Tumor Relapse and Metastasis Promotion. Semin Cancer Biol (2020) 60:351–61. doi: 10.1016/j.semcancer.2019.08.019 31454672

[47] De Angelis ML Francescangeli F La Torre F Zeuner A. Stem Cell Plasticity and Dormancy in the Development of Cancer Therapy Resistance. Front Oncol (2019) 9:626. doi: 10.3389/fonc.2019.00626 31355143 PMC6636659

[48] Hu X Ghisolfi L Keates AC Zhang J Xiang S Lee DK. Induction of Cancer Cell Stemness by Chemotherapy. Cell Cycle (2012) 11(14):2691–8. doi: 10.4161/cc.21021 22732500

[49] Bertolini G Roz L Perego P Tortoreto M Fontanella E Gatti L. Highly Tumorigenic Lung Cancer CD133+ Cells Display Stem-Like Features and are Spared by Cisplatin Treatment. Proc Natl Acad Sci U.S.A. (2009) 106(38):16281–6. doi: 10.1073/pnas.0905653106 PMC2741477 19805294

[50] Chen CP Weinberg VK Jahan TM Jablons DM Yom SS. Implications of Delayed Initiation of Radiotherapy: Accelerated Repopulation After Induction Chemotherapy for Stage III non-Small Cell Lung Cancer. J Thorac Oncol (2011) 6(11):1857–64. doi: 10.1097/JTO.0b013e318229a41e 21964528

[51] El Sharouni SY Kal HB Battermann JJ. Accelerated Regrowth of non-Small-Cell Lung Tumours After Induction Chemotherapy. Br J Cancer (2003) 89(12):2184–9. doi: 10.1038/sj.bjc.6601418 PMC2395273 14676792

[52] Minchiotti G Manco G Parisi S Lago CT Rosa F Persico MG. Structure-Function Analysis of the EGF-CFC Family Member Cripto Identifies Residues Essential for Nodal Signalling. Development (2001) 128(22):4501–10. doi: 10.1242/dev.128.22.4501 11714675

[53] Parisi S D'Andrea D Lago CT Adamson ED Persico MG Minchiotti G. Nodal-Dependent Cripto Signaling Promotes Cardiomyogenesis and Redirects the Neural Fate of Embryonic Stem Cells. J Cell Biol (2003) 163(2):303–14. doi: 10.1083/jcb.200303010 PMC2173524 14581455

