# Supplementary figures and images for: CRIPTO Is a Marker of Chemotherapy-Induced Stem Cell Expansion in Non-Small Cell Lung Cancer
Source: Front Oncol. 2022 Jun 2;12:830873. doi: 10.3389/fonc.2022.830873 (PMC9200964; doi:10.3389/fonc.2022.830873)

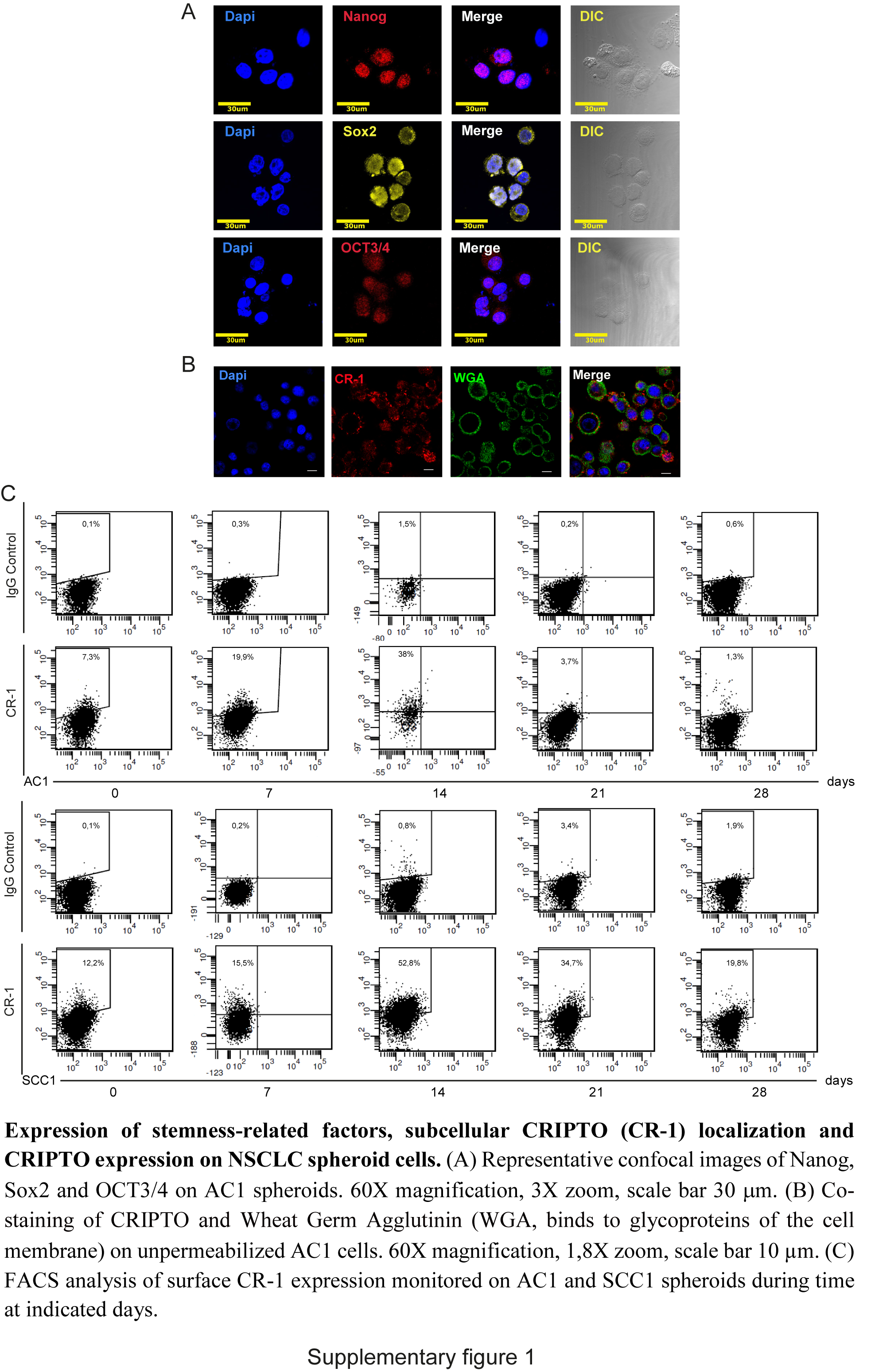

Supplement: Supplementary file 1 [file Image_1.tif]

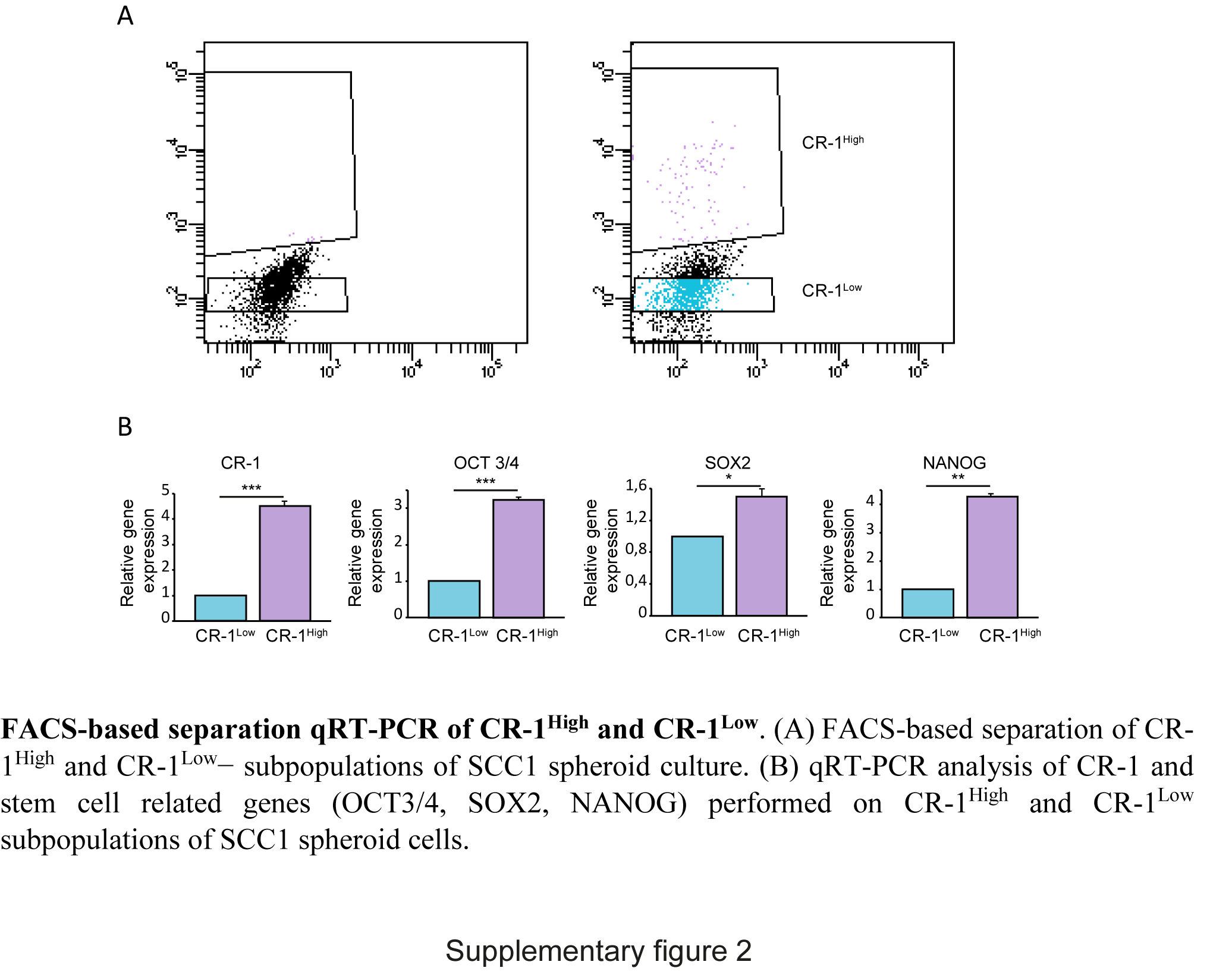

Supplement: Supplementary file 2 [file Image_2.tif]

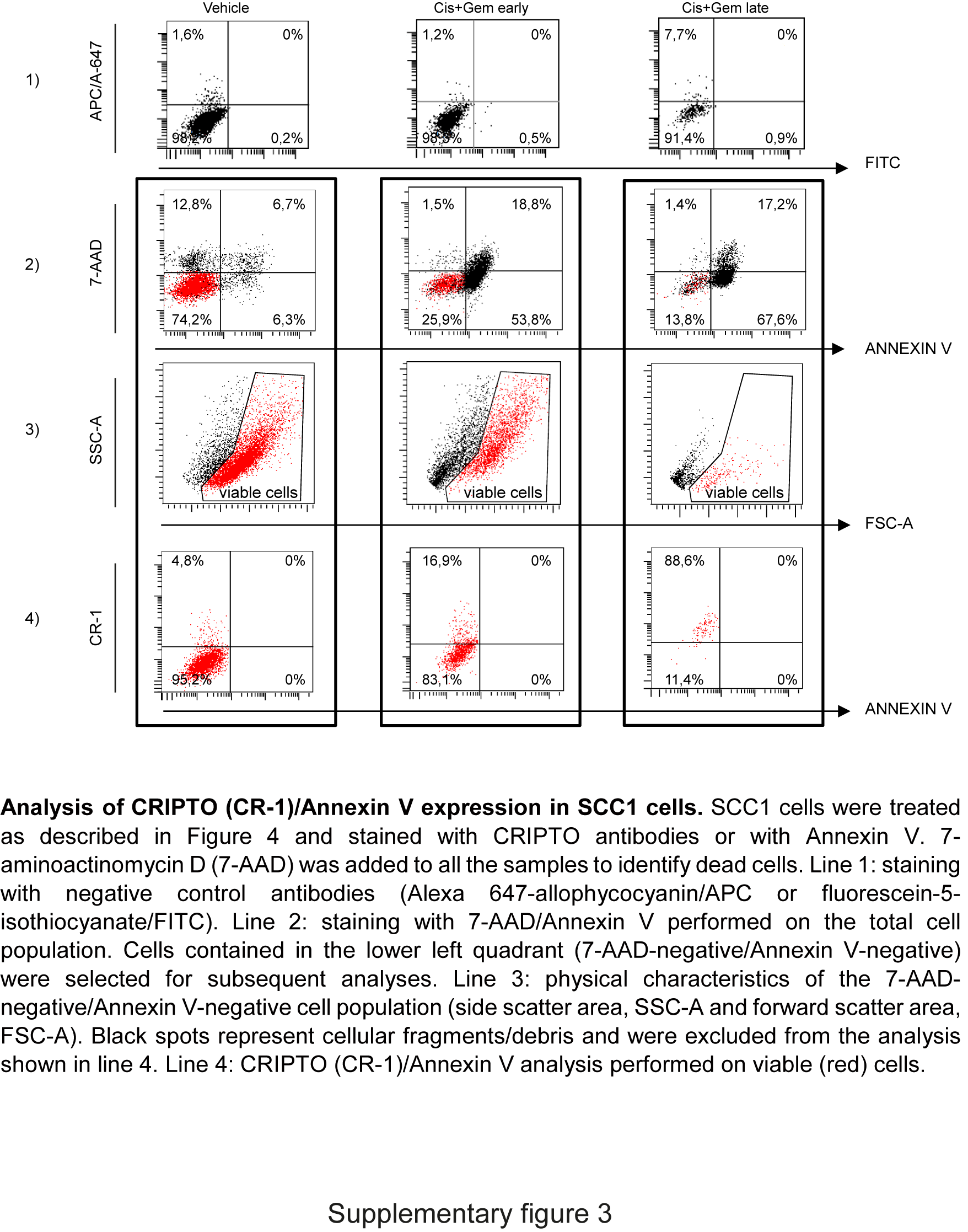

Supplement: Supplementary file 3 [file Image_3.tif]

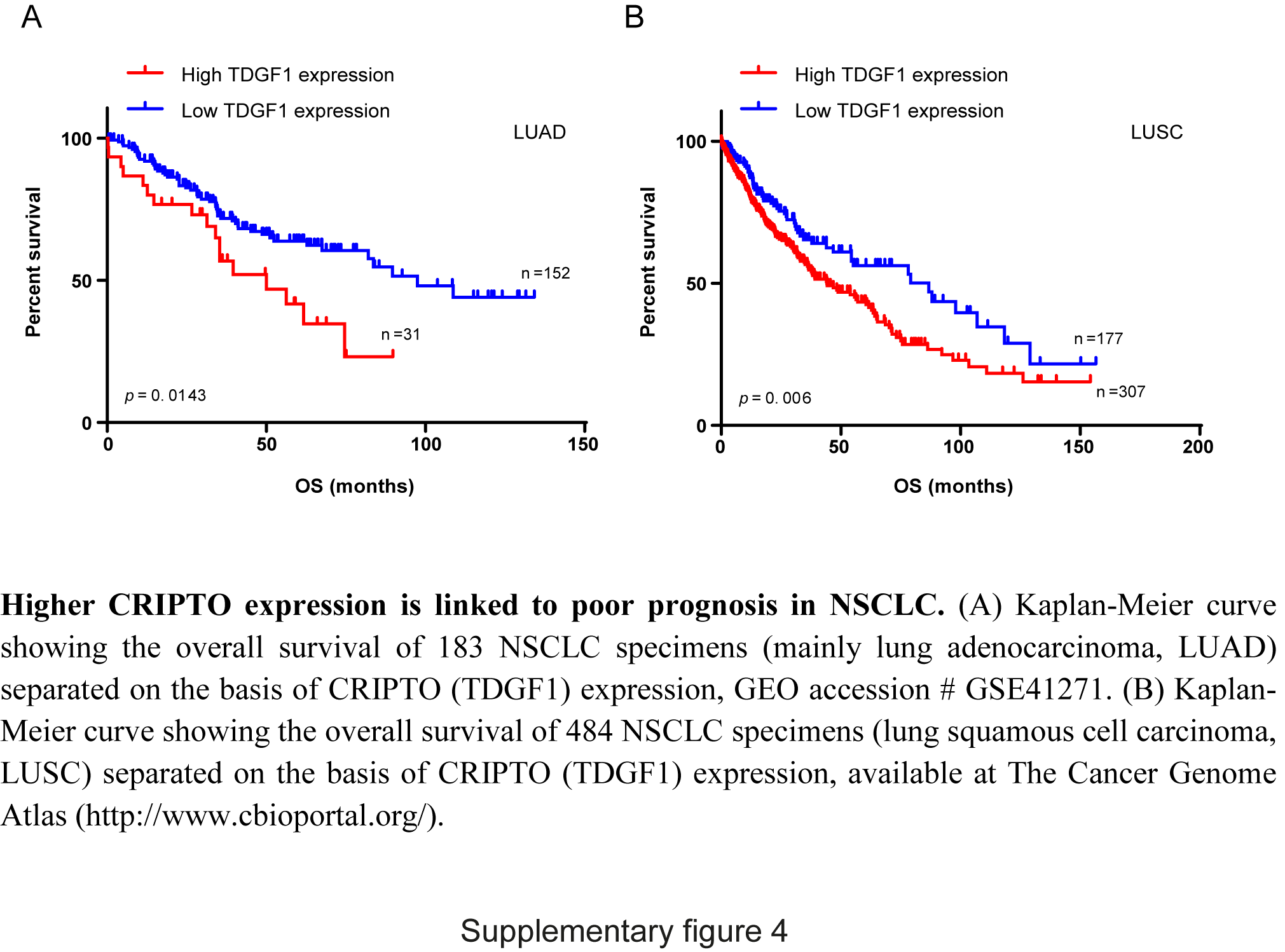

Supplement: Supplementary file 4 [file Image_4.tif]
