# Supplementary material for: CRIPTO Is a Marker of Chemotherapy-Induced Stem Cell Expansion in Non-Small Cell Lung Cancer
Source: Front Oncol. 2022 Jun 2;12:830873. doi: 10.3389/fonc.2022.830873 (PMC9200964; doi:10.3389/fonc.2022.830873)
Supplement: Supplementary file 5 [file Table_1.docx]

| NSCLC  spheroids | Tumor Type | KRAS | EGFR | EML4-ALK fusion | PTEN | PI3K | HER2 |
| --- | --- | --- | --- | --- | --- | --- | --- |
| SCC1 | SCC (pT3pN0pMX IIB-G3) | wt | wt | no | wt | wt | wt |
| SCC2 | SCC (pT2N0 IB) | mut G12C (ggt>tgt) | wt | no | wt | wt | wt |
| AC1 | AC (pT4pN1 IIIA-G3) | mut G12C (ggt>tgt) | wt | no | wt | wt | wt |
| AC2 | AC (pT2pN2pMx IIIA-G2) | wt | wt | no | wt | wt | wt |

| **NSCLC**  **spheroids** | **EGFR gene copy number (mean)** | **CHR7 centromere copy number (mean)** | **Ratio EGFR/CHR7** | **EGFR gene status** | **N of counted cells** |
| --- | --- | --- | --- | --- | --- |
| **SCC1** | 10.3 | 3.71 | 2.77 | Amplification* | 253 |
| **SCC2** | 8.5 | 2.65 | 3.2 | Amplification* | 180 |
| **AC1** | 2.24 | 1.68 | 1.34 | NA*** | 123 |
| **AC2** | 9.2 | 3.26 | 2.82 | Amplification* | 178 |

| **NSCLC**  **spheroids** | **HER2 gene copy number** | **CHR17 centromere copy number** | **Ratio HER2/CHR17** | **HER2 gene status** | **N of counted cells** |
| --- | --- | --- | --- | --- | --- |
| **SCC1** | >10 | 2 | >2 | Amplified | 115 |
| **SCC2** | 3 | 2 | 1.5 | NA*** | 80 |
| **AC1** | 3 | 2 | 1.5 | NA*** | 60 |
| **AC2** | 2 | 2 | 1 | NA*** | 100 |

**Supplementary Table 1.** Clinical and mutational features of NSCLC spheroids. (A) Clinico-pathological and genetic characterization of tumors used to derive NSCLC spheroids; SCC: lung squamous cell carcinoma, AC: lung adenocarcinoma. (B-C) FISH analysis of EGFR (upper panel) and HER2 (lower panel) in NSCLC spheroids.

Supplementary Table 1
